# 32219 Differences in cell death in methionine versus cysteine depletion

**DOI:** 10.1017/cts.2021.429

**Published:** 2021-03-30

**Authors:** Katherine F. Wallis, Isabelle R. Miousse

**Affiliations:** University of Arkansas for Medical Sciences

## Abstract

ABSTRACT IMPACT: Reducing methionine levels has repeatedly been shown to reduce cancer growth in vivo, while at the same time increasing lifespan in healthy animals. However, the mechanisms behind the beneficial effects of methionine restriction are currently unknown. OBJECTIVES/GOALS: We hypothesized that comparing the response of a cancer cell line to depletion of the amino acids methionine and cysteine would give us insight into the critical role of these two closely related amino acids in cancer, and help advance methionine restriction on the translational science spectrum. METHODS/STUDY POPULATION: We used the human melanoma cell line A101D to analyze the response to three conditions: methionine depletion, methionine replacement with homocysteine, and cysteine depletion in vitro. We measured proliferation/viability, gene expression patterns, and glutathione levels. We also assessed ferroptotic versus apoptotic cell death. We then used a normal human fibroblast cell line to compare responses. RESULTS/ANTICIPATED RESULTS: The transcription factors ATF4 and NRF2 were activated by all three tested conditions in melanoma cells. Glutathione levels were decreased by ˜40% in cells grown without methionine, and by 95% in cells grown without cysteine. Lipid peroxidation was increased in cells grown without cysteine, but not in cells grown without methionine. Inhibiting ferroptotic cell death partially rescued proliferation in cysteine-depleted but not in methionine-depleted cells. Almost 70% of cells grown in methionine-depleted media stained positive for Annexin V, an indicator of apoptosis, compared to only 20% of cells grown in cysteine-depleted media. In normal cells, ferrostatin recued proliferation/viability to 86% of control levels in cysteine-depleted cells. Ferrostatin did not affect methionine-depleted normal cells. DISCUSSION/SIGNIFICANCE OF FINDINGS: These results indicate that methionine depletion leads to apoptosis, while cysteine depletion leads to ferroptosis. We found overlapping pathways activated by methionine and cysteine depletion at the gene expression levels, but divergences in cell death pathways ultimately activated.

